# Synergistic Effects of Polyphenols and Stannous Ions on Pellicle Modification and Erosion Protection In Situ

**DOI:** 10.3390/dj13100442

**Published:** 2025-09-26

**Authors:** Jasmin Flemming, Melina Meier, Vanessa Schmitt, Christian Hannig, Matthias Hannig

**Affiliations:** 1 Clinic of Operative Dentistry, Medical Faculty Carl Gustav Carus, Technische Universität Dresden, Fetscherstraße 74, D-01307 Dresden, Germany; melina.meier@uniklinikum-dresden.de (M.M.); christian.hannig@uniklinikum-dresden.de (C.H.); 2 Clinic of Operative Dentistry, Periodontology and Preventive Dentistry, University Hospital, Saarland University, Building 73, D-66421 Homburg, Saar, Germany; vanessa.schmitt@uks.eu (V.S.); matthias.hannig@uks.eu (M.H.)

**Keywords:** fluorides, tannic acid, stannous ions, pellicle, ultrastructure, erosion, polyphenols, biofilms, caries

## Abstract

**Background**: Stannous ions and polyphenols are effective substances in preventive dentistry. The present study’s aim was to investigate whether a combination of these substance groups can achieve increased efficacy. **Methods**: Initial biofilm formation was performed on bovine enamel slabs, carried by 10 subjects intraorally. The subjects rinsed with tannic acid, SnCl_2_, SnF_2_, a combination (50:50) of tannic acid and SnCl_2_, or a combination of tannic acid and SnF_2_, with no rinsing in the negative control. Bacterial adherence, glucan formation (8 h, 48 h oral exposition,) and calcium release kinetics were measured (pH 2; 2.3; 3). Statistics were performed with the Kruskal–Wallis test (*p* < 0.05), Mann–Whitney U test (*p* < 0.05), and Bonferroni–Holm correction. **Results**: All rinsing solutions reduced bacterial adherence by more than 50%. Initial bacterial colonization and glucan formation was significantly reduced by SnF_2_ and SnCl_2_ as well as their combinations with tannic acid. The most significant reductions in calcium release at pH 2; 2.3; and 3 were obtained by SnF_2_ and the combination of SnF_2_ and tannic acid. At the acidic pH 2.0, SnF_2_, SnCl_2_, and tannic acid and SnF_2_ showed significant protection compared to the control (*p* ≤ 0.01). TEM micrographs indicated that rinsing with SnF_2_ and tannic acid leads to pronounced electron dense, thick pellicle layers. **Conclusions**: SnCl_2_ and SnF_2_, as well as their combinations with tannic acid, led to a reduction in initial bacterial colonization and glucan formation, showing an erosion-protective effect. These findings confirm the clinical applicability hitherto suspected by in vitro findings.

## 1. Introduction

Caries and erosive tooth wear have a major impact on all dental hard tissues and oral health in general [1,2]. The benefits from fluorides, especially stannous fluoride, as well as polyphenolic plant extracts, are well-established [3,4,5].

The acquired enamel pellicle provides a physiological barrier against erosive attacks and attachment of bacteria on the enamel surface [2,3]. The pellicle is formed on the tooth surface after contacting saliva and sulcus fluid. It consists of proteins, lipids, and other macromolecules originating from saliva, sulcus fluid, and bacteria [6]. The ultrastructure of the pellicle consists of an electron-dense basal layer on the enamel surface and globular and granular structures that are attached loosely above the basal layer. The acid-resistant effect of the pellicle can be attributed to the basal layer [7].

Plant-based polyphenols modify the composition of the acquired enamel pellicle, as described in ultrastructural investigations [2,8,9,10,11]. It was shown that tannic acid, a high-molecular-weight polyphenol with a pentagalloyl-d-glucose core, has at least five functional groups [12]. It interacts with salivary and pellicle proteins via hydrophobic interactions and hydrogen bonds [12]. Consequently, it can be incorporated into the pellicle, since tannic acid forms large polyphenol–protein aggregates through binding with its functional groups [2,3]. Subsequently, the polyphenol–protein aggregates accumulate and integrate into the pellicle. The ultrastructure of the pellicle is altered and appears more electron-dense and thicker [11]. A previous study described these interactions regarding polyphenols and their role in the pellicle structure [3]. Xi et al. showed that this effect between tannic acid and salivary proteins persists over 24 h [13]. Schestakow et al. even demonstrated that this pellicle thickening effect can be observed after rinsing with tannic acid twice a day for 48 h in situ [14]. Consequently, the erosion-protective potential of the pellicle is enhanced [9,10]. In addition, polyphenols inhibit proteolytic enzymes from saliva [15], matrixmetalloproteinases, α-amylase [16], and glycosyltransferase [17]. Consequently, bacterial glucan formation is reduced [16,18]. Additionally, there are fewer binding sites for specific bacterial adhesion on the tooth surface [10,19]. In addition, rinsing with tannic acid leads to bacteriolysis [13]. Further, tannic acid is registered as a GRAS (Generally Recognized As Safe) food additive, and therefore, as far as present studies show, has no cytotoxic effects on the oral mucosa [20]. Hence, intentional interaction of tannic acid with the in situ pellicle can be applied for caries [19,21] and erosion prophylaxis [9,10].

The erosion-protective effect of fluorides is often referred to as the formation of “protective” calcium fluoride particles, forming a “protective” layer on the tooth surface [22]. Consequently, demineralization should be inhibited and remineralization promoted [23]. However, the formation of those particles is pH-dependent and, so far, has been shown for high fluoride concentrations (12,500 ppm) [24]. In low-fluoride-concentrated rinsing solutions, with concentrations around 500 ppm, no calcium fluoride layer was observed [2].

Compared to other fluoride compounds, preventive measures with stannous ions can prevent the tooth surface significantly from dental erosions [25,26,27]. A previous study described and illustrated the protective nature of stannous fluoride and chloride [28]. In addition, bacterial colonization is also significantly reduced after the use of stannous fluoride and stannous chloride compared to sodium fluoride and sodium monofluorophosphate [29]. Interestingly, the fluoride ion seems to have a minor impact on these effects compared to the bound cation [29].

When comparing the different cations, polyvalent stannous ions catalyze pellicle protein cross-linking to a higher extent than monovalent cations like sodium ions [30]. Therefore, the pellicle appears thicker and more electron-dense in ultrastructural investigations [29]. Ganss et al. showed that stannous ions have a higher affinity to minerals of the enamel surface than to proteins. Hence, stannous ions can be found attached to the enamel surface and form an acid-resistant layer or incorporate into the demineralized layer of the enamel surface [25,27,29]. Very positive results were obtained for erosion prevention, since stannous ions even showed protective effects after intense acid attacks [25,27,31].

Although polyphenols and stannous fluoride and chloride both lead to a more electron-dense and thicker pellicle ultrastructure, their effects on the enamel surface and pellicle layer differ from each other [19,29]. Polyphenols form large protein aggregates that attach to the pellicle layer, followed by pellicle thickening, whereas stannous ions show a higher affinity for the enamel minerals, thereby forming an acid-protective layer and secondarily leading to increased thickness of the pellicle by promoting cross-linking of pellicle proteins.

So far only in vitro studies evaluated a possible effect of polyphenols in combination with fluorides on dental erosion [4,5,31,32]. However, there are no in situ or in vivo studies that have investigated the combined effects on bacterial adhesion, erosive tooth wear, or the ultrastructure of the pellicle.

Since Johannes et al. concluded that preventive solutions containing monosubstances of fluorides provide greater protection against erosive tissue loss when used in situ then used in vitro, one aim of the present study is to investigate synergistic effects of fluoride monosubstances and polyphenolic tannic acid in situ [33].

Further central research questions were

- Is tannic acid or stannous fluoride/chloride more potent in its pellicle modifying effect?
- Is there a difference in the rinsing order if tannic acid or stannous fluoride/chloride is used first?
- Is there a synergistic effect if individuals rinse with a combination of tannic acid and stannous fluoride or chloride?

The null hypothesis of the present study is as follows: There is no difference in the number of adherent bacteria and in calcium release kinetics after rinsing with the different agents compared to the no-rinsing control.

Consequently, the present study aimed to investigate the effect of plant-derived polyphenols and stannous fluoride and chloride erosion-protective capacities of the pellicle layer and on the initial bacterial colonization after 8 and 48 h in situ. The investigation will show if these components are possible ingredients of future mouthrinses and therefore effective agents for clinical application.

## 2. Materials and Methods

### 2.1. Subjects

A number of 10 caries-inactive subjects (aged 22–34 years, 5 women, 5 men) participated in the present in situ study. Visual oral examination was carried out by an experienced dentist. The volunteers were all non-smokers and showed no signs of unrestored carious lesions or periodontitis. The study design was checked and approved by the Ethics Committees of Dresden University (EK 147052013, EK 475112016). The study was conducted at the Policlinic of Operative Dentistry at the Dresden University Hospital, Germany.

The aim of the present study was to investigate whether a combination of stannous ions and polyphenols can achieve increased efficacy regarding initial bacterial colonization, glucan formation, and modification of the ultrastructure of the pellicle to improve their acid-protective properties.

### 2.2. Specimens

Cylindrical enamel specimens were obtained from the facial surfaces of extracted bovine incisors using a 5 mm diameter trepan drill under water cooling (*n* = 796). The cattle were 2 years old and BSE negative. Slabs with structural alterations were excluded. The surfaces of the enamel slabs were polished with wet grinding abrasive paper (320–4000 grit). The enamel specimens for the erosion test series were etched at all sites except for the outer enamel surface for 30 s with 37% phosphoric acid gel (Total Etch etching gel, Ivoclar Vivadent AG, Schaan, Liechtenstein) [33]. Sealing was performed with Optibond FL (Kerr Italia, Scafati, Italy) [33]. Firstly, Optibond Primer was applied for 30 s. Secondly, Optibond Adhesive was applied with a microbrush. Afterwards the slabs were light-cured in a halogen light furnace for 30 s [33]. The application of adhesive followed by light curing was repeated three times. Afterwards, the smear layer was removed by ultrasonication in NaOCl (3%) for 3 min [33]. Subsequently, the slabs were washed for 5 min in distilled water. After this, the specimens were disinfected in ethanol (70%) for another 10 min under ultrasonication. Finally, the samples were washed again and stored in distilled water for 24 h to form a hydration layer before exposure in the oral cavity [33].

### 2.3. Test Solutions

All tested experimental rinsing solutions were prepared freshly from the pure substances before each experiment (Table 1). To prepare the tannic acid mouthrinse solution, 13.6 mg of pure tannic acid was weighed. Following this, 8 mL of bidistilled water was added. The resulting solution was shaken until the solid substance was completely dissolved. The preparation of the stannous fluoride mouthrinse was analogous; here, 16.5 mg were weighed [26,29]. For the stannous chloride mouthrinse, 23.77 mg were weighed and brought into the solution as described above. For the preparation of the combined mouthrinse solution of tannic acid and stannous fluoride or stannous chloride, 8 mL of each of the pure substances was prepared. Afterwards, 4 mL each was filled into a tube and mixed by shaking, so a solution with a mixing ration of 1:1 and a volume of 8 mL was produced. No-rinsing controls were performed for all assays as a comparison [29].

### 2.4. Pellicle Formation In Situ

Individual upper jaw splints were customized for each volunteer for in situ pellicle formation. The splints were built of polymethylmethacrylate and stainless-steel clamps. Small buccal cavities for the insertion of enamel slabs were prepared. Up to six slabs were fixed into the splints at the region of the premolars and first molars. Polyvinylsiloxane impression material was used as material for fixation (Provil novo light regular set, Heraeus Kulzer, Hanau, Germany). Only the enamel surfaces were exposed to the oral cavity [34]. The participants brushed their teeth one hour before wearing the splints without toothpaste. Furthermore, the participants were instructed not to eat or drink during the test period. The investigation took place on different days, so a 48 h washout time was ensured. The laboratory staff were blinded and did not know about the allocation of the experiments [29]. To allow physiological in situ pellicle formation, the splints were carried intraorally for 1 min. A 10 min rinse with the specific test solution (tannic acid, stannous fluoride, stannous chloride, combination of tannic acid and stannous fluoride (ratio 1:1), or combination of tannic acid and stannous chloride (ratio 1:1)) followed [11,29]. The splints were carried intraorally overnight for 8 h to allow initial bacterial colonization. For the erosion testing series, the splints were carried intraorally for 30 min. Unrinsed specimens with a 30 min/8 h in situ pellicle as well as native enamel slabs served as controls. Prior to fluorescence microscopic evaluation or in vitro erosive challenge, test specimens were rinsed with NaCl, which ensured residue-free removal of salivary debris and non-adherent bacteria.

### 2.5. Determination of In Vitro Erosion

After 30 min of intraoral exposure, the test specimens were carefully removed from the splints and embedded in an Eppendorf cup with silicon impression material, followed by incubation of two samples each in hydrochloric acid of pH 2.; 2.3; and 3.0 over a period of 2 min [26]. Continuous circulation during the incubation time was ensured by constant pumping of the 100 µL pipette. Every 15 s, 100 µL of sample solution was removed for photometric analysis. After each extraction, 100 µL of fresh hydrochloric acid of the corresponding pH value was immediately substituted to achieve a constant acid volume of 1 mL [35]. The physiological 30 min pellicle without any rinses and native enamel samples without pellicles served as controls. The determination of the dissociated calcium was performed by using the Arsenazo III method (Calcium AS FS, DiaSys, Diagnostic Systems GmbH, Holzheim, Germany). It forms a bluish-purple complex with the released ions. All measurements were performed as triplicate tests, with the average adsorption being calculated [26].

### 2.6. Microscopy Analyses: DAPI Combined with Concanavalin A (ConA) Staining

DAPI staining was conducted as described previously [29]. The blue, fluorescent DAPI (4′,6-diamidino-2-phenylindole) dye binds mainly to AT-rich nucleotide sequences of double-stranded DNA. DAPI binding to DNA leads to an increase in fluorescence intensity. This fluorescence microscopic method was chosen for calculation of the total bacterial count [29]. The resulting complex is stable at room temperature. After 8 h of intraoral exposure, the enamel samples were carefully removed and rinsed with sodium chloride. Samples were stained for 15 min with 0.75 µL nucleic acid dye in a dark chamber [29]. After the incubation time, the specimens were dried at room temperature and analyzed by epifluorescence microscopy (Axioskop II, Zeiss, Oberkochen, Germany). At 1000-fold magnification, the fluorescence microscopic evaluation was performed using the DAPI light filter (BP 365, FT 395, LP 397) [29]. The adherent bacteria in ten representative microscopic ocular grid fields per sample were counted, with subsequent calculation per square centimeter. The combination of the red fluorescent Concanavalin A (Invitrogen, Molecular probes, Darmstadt, Germany) and the blue fluorescent DAPI staining allows the evaluation of the interaction between bacteria and glucan structures [36,37]. The concanvalin A stock solution (5 mg/mL Concanavalin A—Alexa Fluor 594 in 0.1 M sodium hydrogen phosphate) was stored at −20 °C. The working solution was a 5 µL stock solution in 245 µL buffer solution. The buffer solution contains 1 mM CaCl_2_, 1 mM MgCl_2_, and 1 mM MnCl_2_ in a phosphate-buffered solution [29].

### 2.7. BacLightTM Viability Assay

The LIVE/DEAD BacLightTM Bacterial Viabiltiy Kit (Invitrogen, Molecular probes, Darmstadt, Germany) contains two nucleic acid stains. Using the BacLightTM staining kit, it is possible to differentiate between vital and avital adherent bacteria. The green, fluorescent nucleic acid dye SYTO^®^9 penetrates both vital and avital bacteria with damaged cell membranes. The red fluorescent propidium iodide penetrates only into bacteria with damaged membranes. As a result, dead bacteria fluoresce red. If both dyes are applied at the same time, vital cells appear green and dead cells red under the fluorescence microscope. Similar amounts of component A (Syto9 1.67 mM/propidium iodide 1.67 mM, 300 µL DMSO) and B (Syto9 dye 1.67 mM/propidium iodide 18.3 mM, 300 µL DMSO) were mixed [29]. A volume of 2 µL was pipetted to 1 mL of saline solution [29]. The enamel samples were rinsed with sodium chloride. Afterwards they were incubated in this staining solution for 10 min in a dark chamber. Finally, the samples were rinsed again with sodium chloride and dried at room temperature. Afterwards, the enamel specimens were evaluated with a fluorescence microscope using the FDA filter and the ethidium bromide filter [29].

### 2.8. Ultrastructural Investigation of the In Situ Formed Pellicle (TEM)

To investigate the impact of the different testing solutions on the pellicles’ ultrastructure, transmission electron microscopic investigation was performed. After oral exposure, 2 exemplary 8 h pellicle samples of 2 volunteers of every treatment were evaluated [2]. The enamel slabs were rinsed with distilled water after they had been carefully removed from the splints. The slabs were fixed in 2.5% glutaraldehyde, 1% paraformaldehyde, and 0.1 M cacodylate for 2 h [2]. Subsequently, the test specimens were washed in 0.1 M cacodylate. Postfixation took place in 1% osmium tetroxide for 2 h [2]. Dehydration of the samples was performed by storing them in alcoholic solutions of increasing concentration (50%, 70%, 90%, 100%) [2]. The pellicle site was then embedded with Araldite CY 212 (Plano, Wetzlar, Germany) and the dentin was removed with a diamond bur. Decalcification of the samples took place by storage in 1 M HCl, followed by re-embedding with Araldite [2]. Ultrathin sections of the pellicle samples were cut with a diamond knife, mounted on pioloform-coated copper grids, and contrasted with uranyl acetate and lead citrate. Transmission electron microscopic analysis of the pellicle samples was performed at 3000-to-50,000-fold magnification using TEM TECNAI 12 Biotwin (FEI Eindhoven, The Netherlands) [2].

### 2.9. Statistics

Samples size calculation was performed with BioMath (Rostock, Germany) and was determined based on a previously performed study with the same design [38]. Statistical evaluations of the fluorescence microscopic data as well as the calcium and phosphate data were carried out using the Kruskal–Wallis and the Mann–Whitney U tests, since the data was not normally distributed (Kolmogorov–Smirnov test). Afterwards, a Bonferroni–Holm correction took place (*p* ≤ 0.05). The statistical analysis was performed using the Software SPSS 27.0.1.0 (IBM, Ehningen, Germany).

## 3. Results

In the present in situ study, initial bacterial colonization was evaluated by DAPI staining and a BacLight fluorescence staining method after rinsing with stannous fluoride, stannous chloride, and tannic acid, as well as the combinations of tannic acid + stannous chloride or tannic acid + stannous fluoride, and the successive rinsing of one rinsing solution after the other. Additionally, glucan structures were evaluated by Concanavalin A staining (Con A). Overall, the rinses containing stannous fluoride, or the combination of tannic acid and stannous fluoride, outperformed the other groups. The combination showed a high substantivity on the tooth surface, with sustainable effects after 48 h.

### 3.1. Fluorescence Microscopic Assays

#### 3.1.1. DAPI Staining; 8 h Oral Exposition

**Figure 1:**
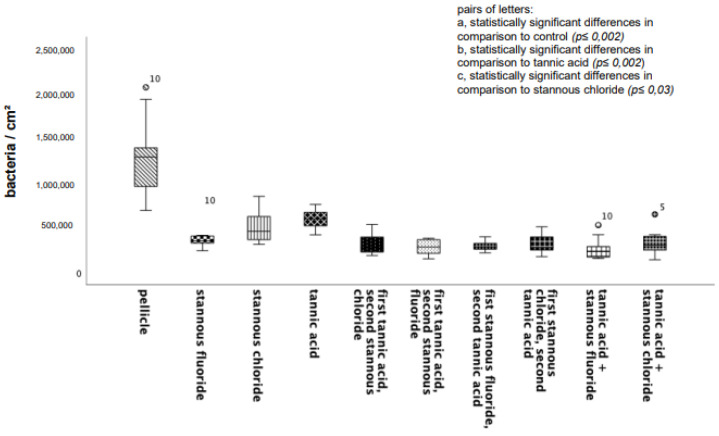
Boxplot diagram. Influence of rinsing with stannous ions containing solutions, tannic acid, and combinations of these solutions on initial bacterial adhesion to enamel bovine slabs in situ over 8 h. Slabs without rinsing served as a negative control (pellicle). Evaluation of the bacterial colonization with the DAPI method. Columns marked with the same pair of letters are significantly different (Mann–Whitney U test followed by Bonferroni–Holm correction, *p* < 0.002–0.03); *n* = 200 bovine samples.

Compared to the control specimens, all tested mouthrinses achieved a significant reduction in adherent bacteria. The number of adherent microorganisms was lowest after rinsing with the combination of tannic acid and stannous fluoride (Figure 1).

#### 3.1.2. Concanavalin A Staining; 8 h Oral Exposition

**Figure 2:**
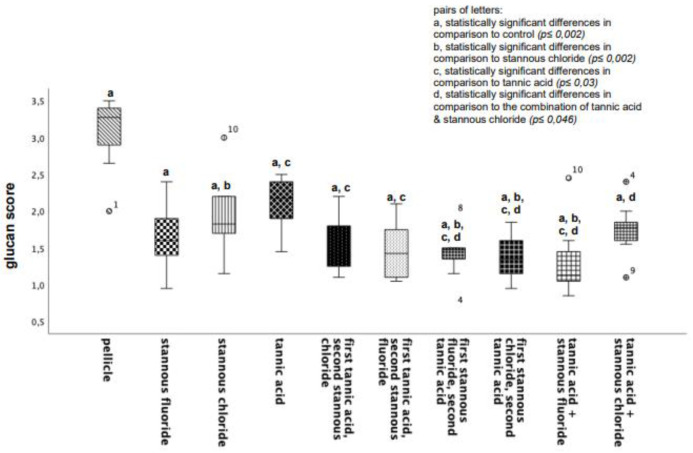
Boxplot diagram. Influence of rinsing with stannous ions containing solutions, tannic acid, and combinations of these solutions on glucan synthesis in situ over 8 h. Slabs without rinsing served as a negative control (pellicle). Evaluation of glucan synthesis was performed with the Con A staining method. Columns marked with the same pair of letters are significantly different (Mann–Whitney U test followed by Bonferroni–Holm correction, *p* < 0.002–0.046); *n* = 200 bovine samples.

**Figure 3:**
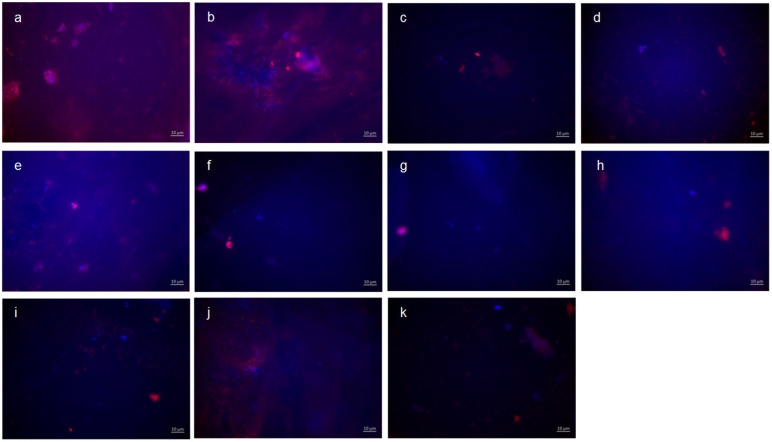
Fluorescence microscopy, combination of DAPI and Con A staining: (**a**,**b**) control without rinsing; (**c**) stannous chloride; (**d**) stannous fluoride; (**e**) tannic acid; (**f**) stannous chloride first, tannic acid second; (**g**) stannous fluoride first, tannic acid second; (**h**) tannic acid first, stannous chloride second; (**i**) tannic acid first, stannous fluoride second; (**j**) combination of tannic acid and stannous chloride; and (**k**) combination of tannic acid and stannous fluoride. Scale bar in the right corner: 10 µm.

All tested experimental solutions showed a significant reduction in the glucan formation in comparison to the control group. Rinsing with the combination of tannic acid and stannous fluoride resulted in the greatest reduction in glucan structures (Figure 2 and Figure 3).

Representative images visualize bacterial colonization and glucan synthesis on the bovine enamel surface after 8 h of oral exposition (Figure 3). Based on these, blue DAPI staining indicates adherent bacteria at the pellicle layer as well as bacteria–glucan agglomerates in combination with the glucan staining (Con A—red). (A) and (b) show images after 8 h oral exposition without rinsing. Red fluorescent glucan ring structures surrounding blue, fluorescent bacteria are clearly visible (a). Furthermore, bacterial accumulations were surrounded by diffuse glucan clouds (b). A significant reduction in adherent bacteria and glucan structures can be detected after rinsing with stannous chloride, stannous fluoride, and tannic acid (c–e). In addition, small bacterial clusters show distinct glucan structures (c,d). The successive application of the monosubstances (f–i) leads to a further reduction in the bacterial adherence tp the tooth surface. The greatest reduction in adherent bacteria was achieved by the combined rinsing with tannic acid and stannous fluoride (k). As such, no glucan structures were detectable (k).

#### 3.1.3. BacLight Staining; 8 h Oral Exposition

**Figure 4:**
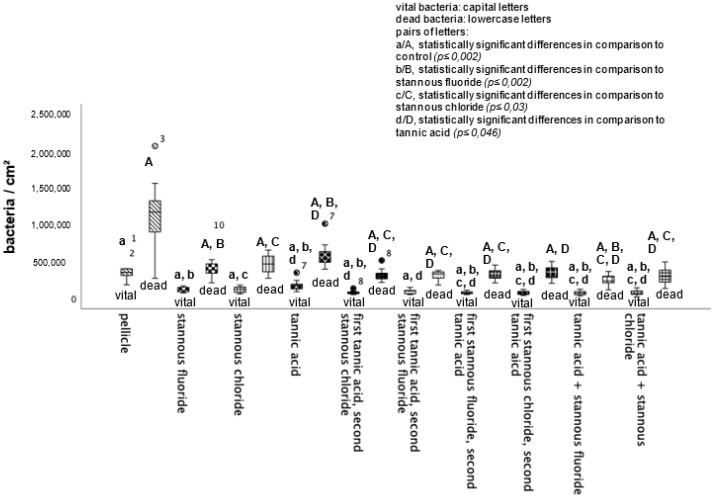
Boxplot diagram. Influence of rinsing with stannous ions containing solutions, tannic acid, and combinations of these solutions on initial bacterial colonization in situ over 8 h. Slabs without rinsing served as a negative control (pellicle). Evaluation of vital and dead bacteria with the BacLight method. Columns marked with the same pair of letters are significantly different (Mann–Whitney U test followed by Bonferroni–Holm correction, *p* < 0.002–0.046); *n* = 200 bovine samples.

BacLight live/dead staining enables a clear differentiation of vital (green) and dead (red) bacteria. Compared to the control, all tested mouthrinses achieved a significant reduction in vital and dead bacteria (Figure 4). The greatest reduction in vital bacteria was obtained by successive application of the mouthrinse of stannous chloride first and tannic acid second. Further, the most significant reduction in dead bacteria was detectable after the rinse with the combination of tannic acid and stannous fluoride (Figure 4).

**Figure 5:**
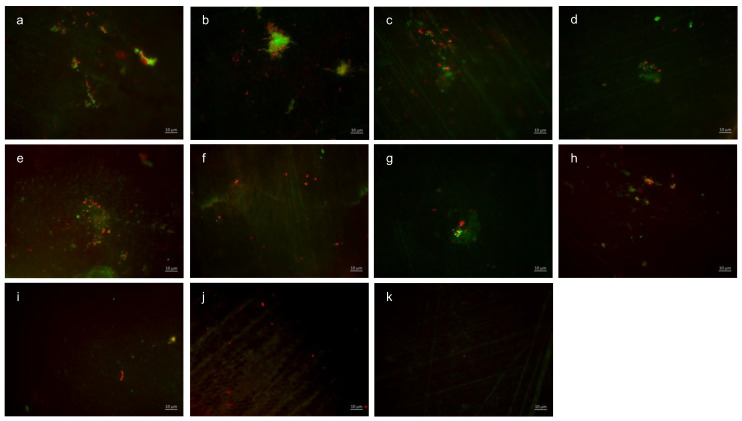
Fluorescence microscopy, BacLight staining: (**a**,**b**) control without rinsing; (**c**) stannous chloride; (**d**) stannous fluoride; (**e**) tannic acid; (**f**) stannous chloride first, tannic acid second; (**g**) stannous fluoride first, tannic acid second; (**h**) tannic acid first, stannous chloride second; (**i**) tannic acid first, stannous fluoride second; (**j**) combination of tannic acid and stannous chloride; and (**k**) combination of tannic acid and stannous fluoride. Scale bar in the right corner: 10 µm.

Representative images visualize bacterial colonization on the bovine enamel surface after 8 h of oral exposition. Thereby, BacLight staining indicates adherent vital (green) and dead (red) bacteria at the pellicle layer (Figure 5). (A) and (b) show images after 8 h oral exposition without rinsing. Thereby, areas with great bacterial accumulations and a large number of vital, green bacteria (b) are characteristic for the control groups without rinsing. A significant reduction in vital and dead adherent bacteria was obtained after rinsing with stannous chloride, stannous fluoride, and tannic acid (c–e). The successive application of the monosubstances (f–i) leads to a further reduction in the bacterial adherence to the tooth surface. The greatest reduction in adherent vital and dead bacteria was achieved by the combined rinsing with tannic acid and stannous fluoride (j,k). As a result, only a few individual dead bacterial cells remained (k).

#### 3.1.4. DAPI Staining; 48 h Oral Exposition

Since rinsing with the combination tannic acid and stannous fluoride showed a significantly reduced number of adherent bacteria after 8 h of oral exposure, the 48-h exposition testing took place with both combinations—tannic acid and stannous chloride as well as tannic acid and stannous fluoride.

**Figure 6:**
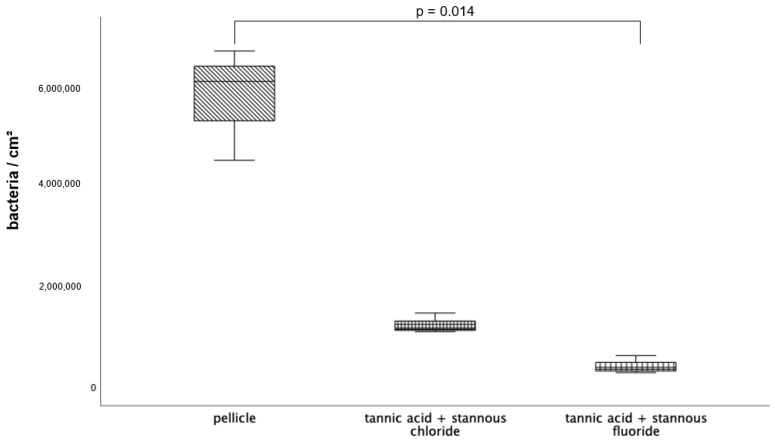
Evaluation of the bacterial colonization with the DAPI method; boxplot diagram. Influence of rinsing with the combinations of tannic acid and stannous chloride and tannic acid and stannous fluoride on initial bacterial adhesion to enamel bovine slabs in situ over 8 h. Slabs without rinsing served as a negative control (pellicle). Columns marked with a parenthesis are significantly different (Mann–Whitney U test followed by Bonferroni–Holm correction, *p* < 0.015); *n* = 18 bovine samples.

**Figure 7:**
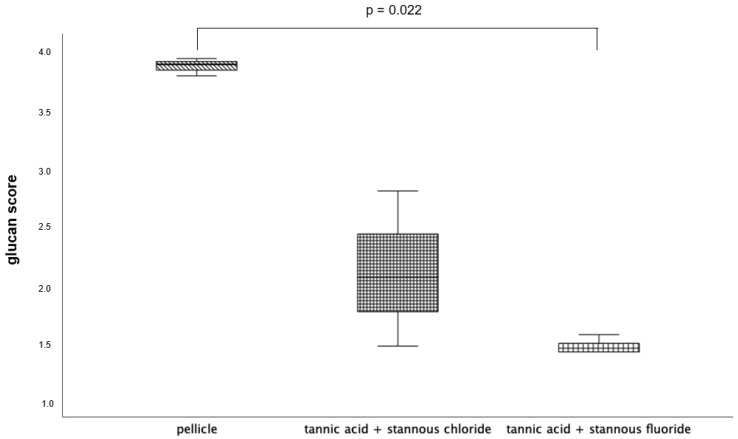
Evaluation of the glucan synthesis, performed with the Con A staining method, presented in a boxplot diagram. Influence of rinsing with the combinations of tannic acid and stannous chloride and tannic acid and stannous fluoride on initial bacterial adhesion to enamel bovine slabs in situ over 48 h. Slabs without rinsing served as a negative control (pellicle). Columns marked with a parenthesis are significantly different (Mann–Whitney U test followed by Bonferroni–Holm correction, *p* < 0.023); *n* = 18 bovine samples.

**Figure 8:**
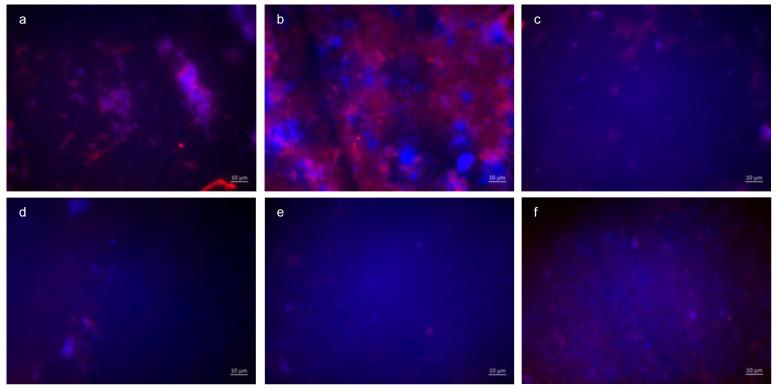
Fluorescence microscopy, combination of DAPI and Con A staining: (**a**,**b**) control without rinsing, (**c**,**d**) combination of tannic acid and stannous chloride, and (**e**,**f**) combination of tannic acid and stannous fluoride. Representative images visualize bacterial colonization and glucan synthesis on the bovine enamel surface after 48 h of oral exposition. Scale bar in the right corner: 10 µm.

The visualization and quantification of oral microorganisms was also performed using DAPI combined with Concanavalin A staining (Figure 6, Figure 7 and Figure 8). In the control without rinsing, an overall distribution of bacteria in large aggregates was detectable. In certain areas, the enamel specimens were completely covered by microbial colonies. Concanavalin A staining showed distinct glucan structures around adherent bacteria in the control without rinsing. No bacteria without glucan structures were detectable. The combination of tannic acid and stannous fluoride significantly reduced bacterial colonization as well as glucan formation.

#### 3.1.5. Concanavalin A Staining; 48 h Oral Exposition

The evaluation (Figure 7) and visualization (Figure 8) of glucan structures was also performed using Concanavalin A staining.

#### 3.1.6. BacLight Staining; 48 h Oral Exposition

**Figure 9:**
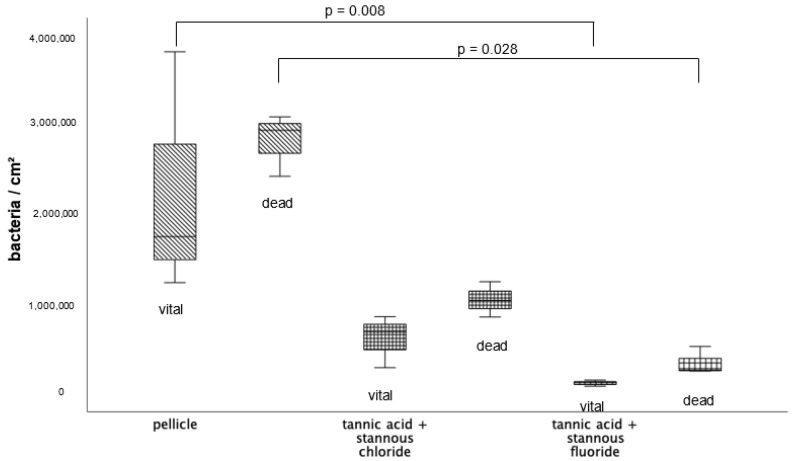
BacLight staining method boxplot diagram. Influence of rinsing with the combinations of tannic acid and stannous fluoride and tannic acid and stannous chloride on initial bacterial colonization in situ over 48 h. Slabs without rinsing served as a negative control (pellicle). Evaluation of vital and dead bacteria with the BacLight method. Columns marked with a parenthesis are significantly different (Mann–Whitney U test followed by Bonferroni–Holm correction, *p* < 0.029); *n* = 18 bovine samples.

**Figure 10:**
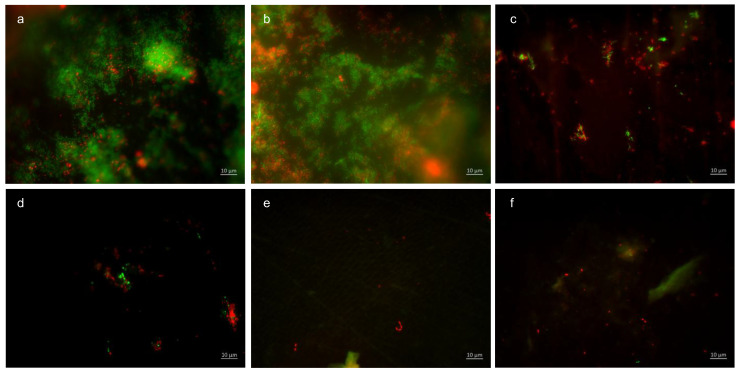
Fluorescence microscopy, BacLight staining method: (**a**,**b**) control without rinsing, (**c**,**d**) combination of tannic acid and stannous chloride, and (**e**,**f**) combination of tannic acid and stannous fluoride. Scale bar in the right corner: 10 µm.

The quantification (Figure 9) and differentiation of vital (green) and dead (red) bacteria (Figure 10) after an oral exposure time of 48 h has also been carried out by using the BacLight live/dead staining. The control without rinsing showed an overall bacterial layer on the slabs. The number of dead bacteria predominated, but a very high number of vital microorganisms were detectable. The predominantly coccoid and rod-shaped bacteria were present in large colonies. Compared to the control, the combination of tannic acid and stannous fluoride achieved a significant reduction in both vital and dead bacteria.

Representative images visualize bacterial colonization on the bovine enamel surface after 48 h of oral exposition. Red and green BacLight staining indicates vital (green) and dead (red) adherent bacteria at the pellicle layer. (A) and (b) show images after 48 h oral exposition without rinsing. Vital and dead fluorescent bacteria are clearly visible as bacterial colonies (a,b).

A significant reduction in adherent bacterial cells can be detected after rinsing with the combined rinsing solution of tannic acid and stannous chloride (c,d). The microorganisms are arranged as small aggregates or individual cells (c,d). The greatest reduction in adherent vital and dead bacteria was achieved by the combined rinsing with tannic acid and stannous chloride (c,d). Thereby, microorganisms are spread out and arranged in aggregates (c,d). Hardly any vital bacterial cells were detectable after rinsing with tannic acid and stannous fluoride (e,f). A great reduction in dead microorganisms was detectable. These cells were visible as individual cells (e,f).

### 3.2. Dissociation of Calcium Ions

The demineralizing-protective effect of the rinsing solutions stannous chloride, stannous fluoride, and tannic acid, as well as the combinations of tannic acid + stannous chloride and tannic acid + stannous fluoride, was evaluated. Specimens without and with pellicles served as controls. In terms of significance, pairwise comparison of mean mineral loss after 120 s of incubation in HCl revealed notable differences. 

#### 3.2.1. Calcium Release 120 s, pH 2.0

**Figure 11:**
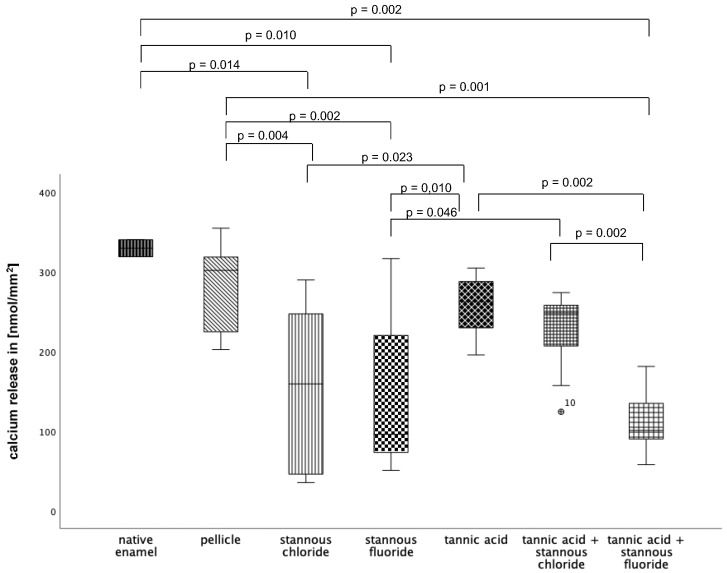
Cumulative calcium release of enamel slabs after 30 min of pellicle formation with application of stannous chloride, stannous fluoride, tannic acid, and the combinations of tannic acid + stannous chloride and tannic acid + stannous fluoride in situ followed by incubation in HCl (pH 2.0) for 120 s. Specimens without and with pellicles served as controls. Significantly different data is marked with parenthesis. At pH 2.0, pellicle formation did not reduce calcium release significantly. A significant reduction in calcium release was observed after rinsing with stannous chloride, stannous fluoride, and the combination of tannic acid + stannous fluoride compared to the native enamel and the pellicle control (*p* ≤ 0.01). The greatest reduction in calcium loss was achieved by stannous fluoride and the combination of tannic acid + stannous fluoride (Kruskal–Wallis test, Mann–Whitney U test, and Bonferroni–Holm correction); *n* = 120 bovine samples.

At pH 2.0 (Figure 11), pellicle formation did not reduce calcium release significantly. Comparing all tested substances, pure tannic acid mouthrinse seems to have the least pronounced impact on the physiological pellicle. A significant reduction in calcium release was observed after rinsing with stannous chloride, stannous fluoride, and the combination of tannic acid + stannous fluoride compared to the native enamel and the pellicle control (*p* ≤ 0.01). The most pronounced reduction in calcium loss was achieved by stannous fluoride and the combination of tannic acid + stannous fluoride (Figure 11).

#### 3.2.2. Calcium Release 120 s, pH 2.3

**Figure 12:**
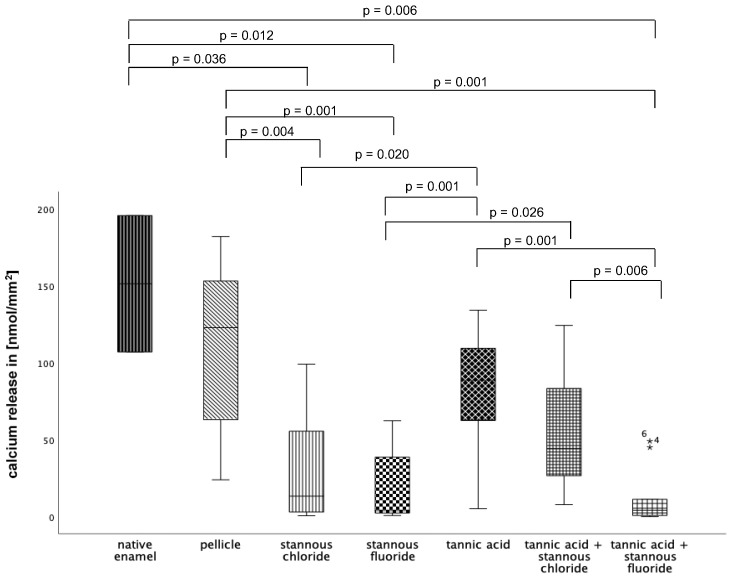
Cumulative calcium release of enamel slabs after 30 min of pellicle formation with application of stannous chloride, stannous fluoride tannic acid, and the combinations of tannic acid + stannous chloride and tannic acid + stannous fluoride in situ followed by incubation in HCl (pH 2.3) for 120 s. Specimens without and with pellicle served as controls. Significantly different data is marked with parenthesis. At pH 2.3, pellicle formation did not reduce calcium release significantly. A significant reduction in calcium release was observed after rinsing with stannous chloride, stannous fluoride, and the combination of tannic acid + stannous fluoride compared to the native enamel and the pellicle control (*p* ≤ 0.036) The greatest reduction in calcium loss was achieved by stannous fluoride and the combination of tannic acid + stannous fluoride (Kruskal–Wallis test, Mann–Whitney U test, and Bonferroni–Holm correction); *n* = 120 bovine samples.

At pH 2.3 (Figure 12), pellicle formation did not reduce calcium release significantly. Comparing all tested substances, pure tannic acid mouthrinse seems to have the least pronounced impact on the physiological pellicle. A significant reduction in cal-cium release was observed after rinsing with stannous chloride, stannous fluoride, and the combination of tannic acid + stannous fluoride compared to the native enamel and the pellicle control (*p* ≤ 0.01). The most pronounced reduction in calcium loss was achieved by stannous fluoride and the combination of tannic acid + stannous fluoride (Figure 12).

#### 3.2.3. Calcium Release 120 s, pH 3.0

**Figure 13:**
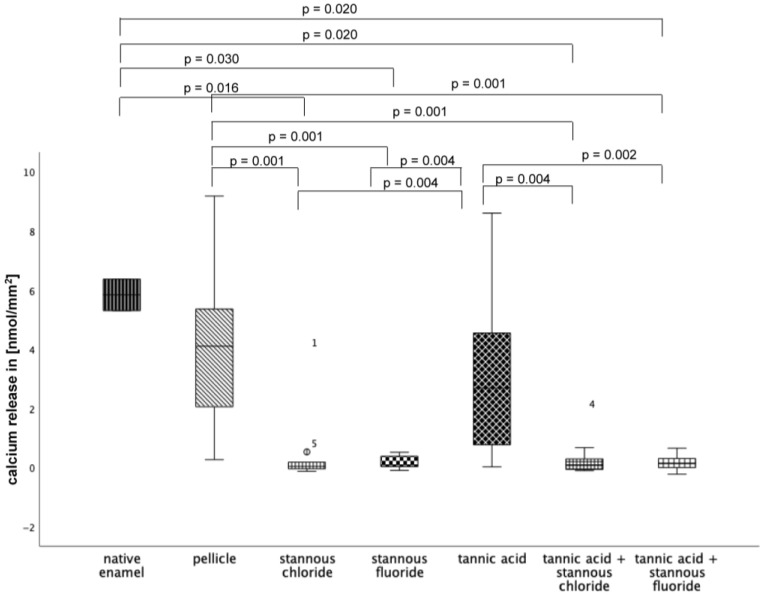
Cumulative calcium release of enamel slabs after 30 min of pellicle formation with application of stannous chloride, stannous fluoride, tannic acid, and the combinations of tannic acid + stannous chloride and tannic acid + stannous fluoride in situ followed by incubation in HCl (pH 3.0) for 120 s. Specimens without and with pellicle served as controls Significantly different data is marked with parenthesis. At pH 3.0, pellicle formation did not reduce calcium release significantly. A significant reduction in calcium release was observed after rinsing with stannous chloride, stannous fluoride, and the combination of tannic acid and stannous chloride, as well as the combination of tannic acid + stannous fluoride, compared to the native enamel and the pellicle control (*p* ≤ 0.03). The greatest reduction in calcium loss was achieved by stannous chloride, stannous fluoride, the combination of tannic acid + stannous chloride, and the combination of tannic acid + stannous fluoride (Kruskal–Wallis test, Mann–Whitney U test, and Bonferroni–Holm correction); *n* = 120 bovine samples.

At pH 3, the greatest reduction in calcium loss was achieved by stannous chloride, stannous fluoride, the combination of tannic acid + stannous chloride, and the combination of tannic acid + stannous fluoride (Figure 13).

### 3.3. Transmission Electron Microscopy

**Figure 14:**
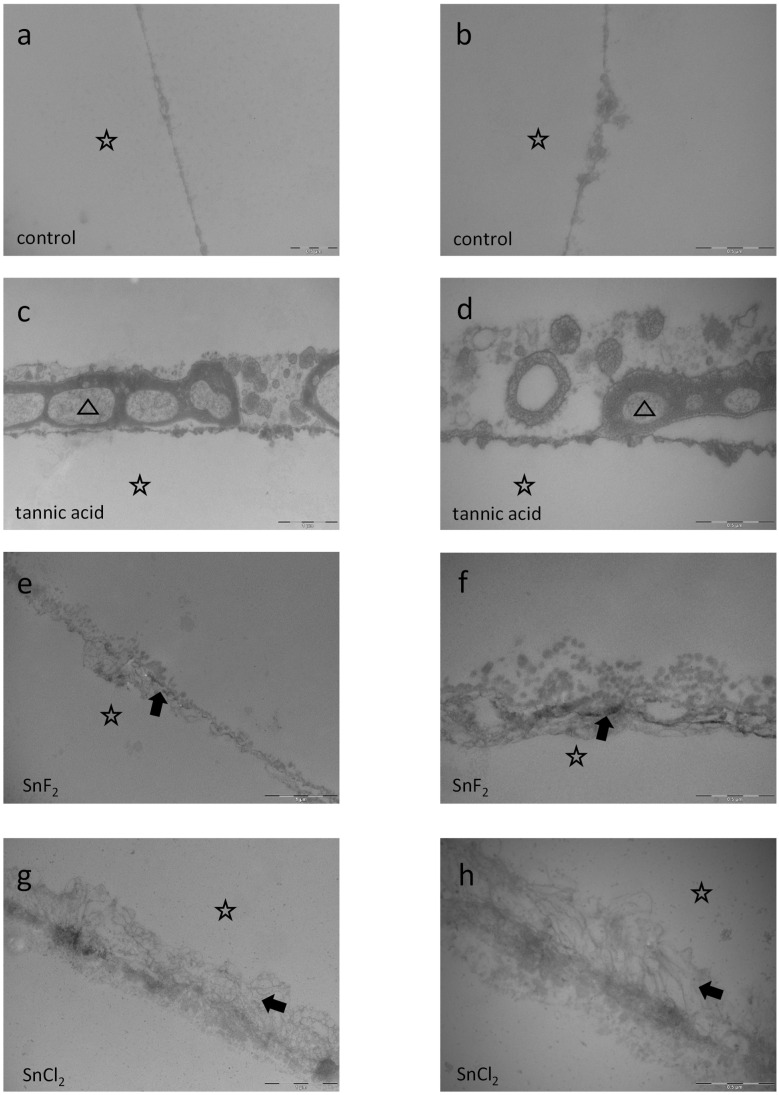
Representative TEM images visualize the pellicle’s ultrastructure. Intraoral exposure time: 8 h. All pellicles show an electron-dense basal layer. This basal layer is covered by varying less electron-dense granular and globular layers (**a**,**b**) Magnification: 30,000- and 49,000-fold; control samples without rinsing show a typical pellicle layer consisting of a thin basal layer and above granular and globular layers (**c**,**d**) Magnification: 18,500- and 49,000-fold; tannic acid rinsing for 10 min, pH 3.9. The treatment with 10 min tannic acid rinsing not only leads to formation of thicker pellicles but also to increased density and inclusion of micellar structures of salivary origin (triangle). (**e**,**f**) Magnification 23,000- and 49,000-fold; rinsing with stannous fluoride for 1 min, pH 4.5. Rinsing with stannous fluoride forms a pellicle layer with an increased amount of globular pellicle compounds. Dark spots indicate stannous ions from the stannous fluoride solution incorporated into the pellicle layer (arrow). (**g**,**h**) Magnification 23,000- and 49,000-fold; rinsing with stannous chloride for 1 min, pH 2.5. The TEM images show typical protein infiltrations below the pellicles’ basal layer due to the low pH of the stannous chloride solution at pH 2.5 (arrow). The former enamel site is marked by a hollow star. Scale bar in the right corner: (**a**,**b**,**d**,**f**,**h**) 0.5 µm; (**c**,**e**,**g**) 1 µm.

**Figure 15:**
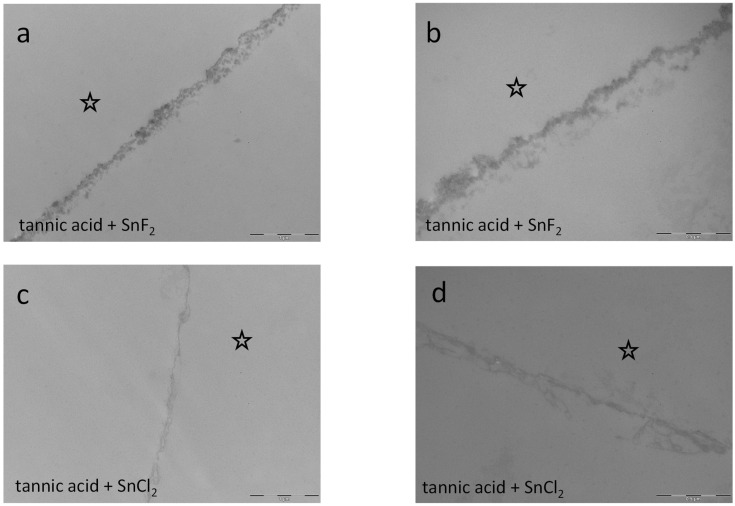
Representative TEM images visualize the pellicle’s ultrastructure. Intraoral exposure time: 8 h. (**a**,**b**) Magnification 23,000- and 49,000-fold; rinsing with the combination of tannic acid and stannous fluoride for 10 min, pH 3.2. The combination of tannic acid and stannous fluoride seems to neutralize their single effects on the pellicle layer. Compared to single rinsing with tannic acid or stannous fluoride, the pellicle is of lower density and thickness (Figure 14c–f). (**c**,**d**) Magnification 23,000- and 49,000-fold; rinsing with tannic acid and stannous chloride for 10 min. pH 2.45. The combination of tannic acid and stannous chloride seems to neutralize their single effects on the pellicle layer. Compared to single rinsing with tannic acid or stannous fluoride, the pellicle is of a lower density and thickness. Additionally, the combination of tannic acid and stannous fluoride seems to form a thicker pellicle layer (**a**,**b**) than the combination of tannic acid and stannous chloride (**c**,**d**). The former enamel site is marked by a hollow star. Scale bar in the right corner: (**a**,**c**): 0.5 µm; (**b**,**d**): 1 µm.

**Figure 16:**
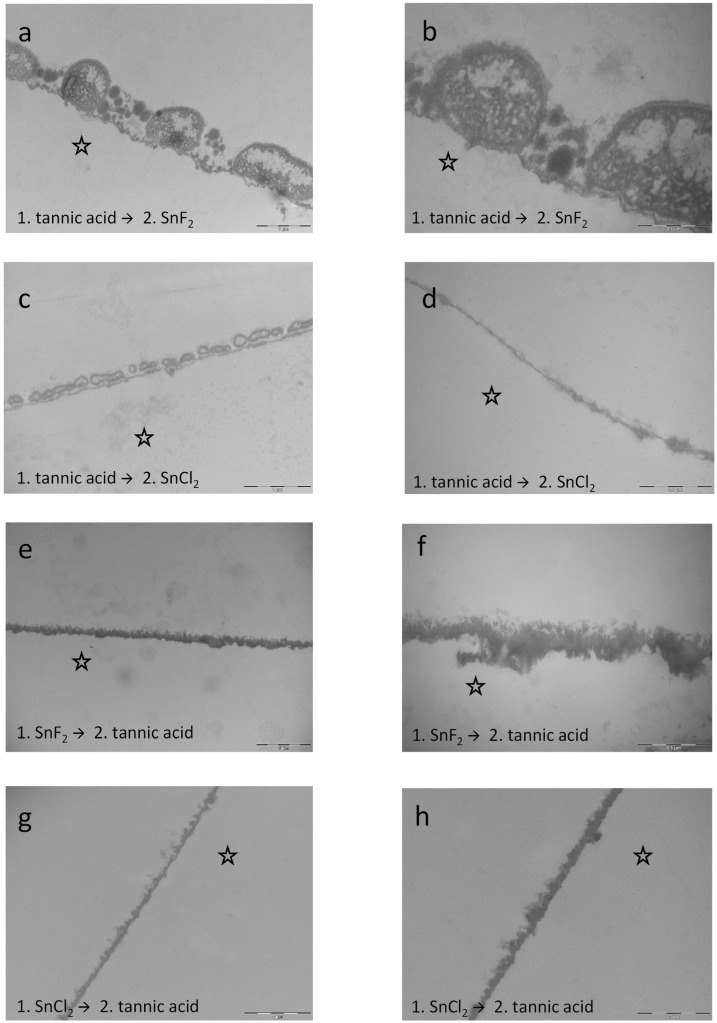
Representative TEM images visualize the pellicle’s ultrastructure. Intraoral exposure time: 8 h. (**a**,**b**) Magnification 23,000- and 49,000-fold; rinsing with tannic acid for 10 min followed by stannous fluoride for 1 min leads to significant effects on the modification of the pellicle layer. The pellicle appears more electron-dense and thicker compared to the control samples. (**c**,**d**) Magnification 23,000- and 49,000-fold; rinsing with tannic acid for 10 min followed by stannous chloride for 1 min. (**e**,**f**) Magnification 18,500- and 49,000-fold; rinsing with stannous fluoride for 1 min followed by tannic acid for 10 min. This subsequent rinsing leads to a distinct modification of the pellicle layer. Compared to the other combinations, the pellicle was massively electron-dense. Incorporation of stannous ions first and the effects of the tannic acid rinsing second resulted in the most intense density in the present experiments. In addition, rinsing with tannic acid second had intense tanning effects at the pellicle layer. (**g**,**h**) Magnification 23,000- and 49,000-fold; rinsing with stannous chloride for 1 min followed by tannic acid for 10 min. The subsequent rinsing leads to an offsetting of the negative effects from stannous chloride rinsing. No protein infiltrations were observed below the pellicle layer. Since the stannous chloride solution still had a pH 2.5, rinsing with tannic acid seems to nullify the demineralizing effect of the stannous chloride solution. The former enamel site is marked by a hollow star. Scale bar in the right corner: (**a**,**c**,**e**,**g**): 0.5 µm; (**b**,**d**,**f**,**h**): 1 µm.

The pellicles’ ultrastructure was investigated after rinsing with tannic acid (Figure 14c,d), stannous fluoride (Figure 14e,f), stannous chloride (Figure 14g,h), the combination of tannic acid and stannous fluoride (Figure 15a,b), and the combination of tannic acid and stannous chloride (Figure 15c,d), as well as after subsequent rinsing with tannic acid for 10 min followed by stannous fluoride for 1 min (Figure 16a,b), tannic acid for 10 min followed by stannous chloride for 1 min (Figure 16c,d), stannous fluoride for 1 min followed by tannic acid for 10 min (Figure 16e,f), and stannous chloride for 1 min followed by tannic acid for 10 min (Figure 16g,h). The intraoral exposure time was 8 h in total. In addition, TEM analyses were conducted on exemplary in situ pellicle samples after 8 h of oral exposition without rinsing, which served as controls (Figure 14a,b). As expected, the physiological 8 h in situ pellicle samples showed the characteristic thin but electron-dense and continuous protein accumulation on the former enamel surface (Figure 14a,b). In comparison, rinsing with tannic acid led to the formation of thicker pellicles but also to increased density and inclusion of bacterial cells (Figure 14c,d). Due to the low pH, rinsing with stannous fluoride and stannous chloride results in typical protein infiltrations (Figure 14e–h). These stannous ion-containing solutions form a pellicle layer with an increased amount of globular pellicle compounds. Dark spots indicate incorporated stannous ions inside the pellicle layer (Figure 14e,f).

Combined rinsing with tannic acid and stannous fluoride neutralizes the above-described effects of the single solutions since the pellicle layer is less thick and electron-dense. A similar effect was investigated for the combination of tannic acid and stannous chloride. When both combinations are compared, the combination of tannic acid and stannous fluoride seems to form a thicker pellicle layer (Figure 15a,b) than the combination of tannic acid and stannous chloride (Figure 15c,d).

In contrast to the combinations, subsequent rinsing showed stronger results. Specifically, rinsing with stannous fluoride first and tannic acid afterwards leads to a distinct modification of the pellicle layer. Compared to the other combinations and rinsing sequences (Figure 14 and Figure 16), the pellicle was massively electron-dense, and the most intense density in the present experiments was detected. In addition, rinsing with tannic acid second had intense tanning effects on the pellicle layer (Figure 16e,f). Further, rinsing with stannous chloride first and tannic acid afterwards leads to an offsetting of the negative effects from the stannous chloride rinsing. No protein infiltrations were observed below the pellicle layer. Since the stannous chloride solution still had a pH 2.5, rinsing with tannic acid seems to nullify the demineralizing effect of the stannous chloride solution (Figure 16g,h).

**Figure 17:**
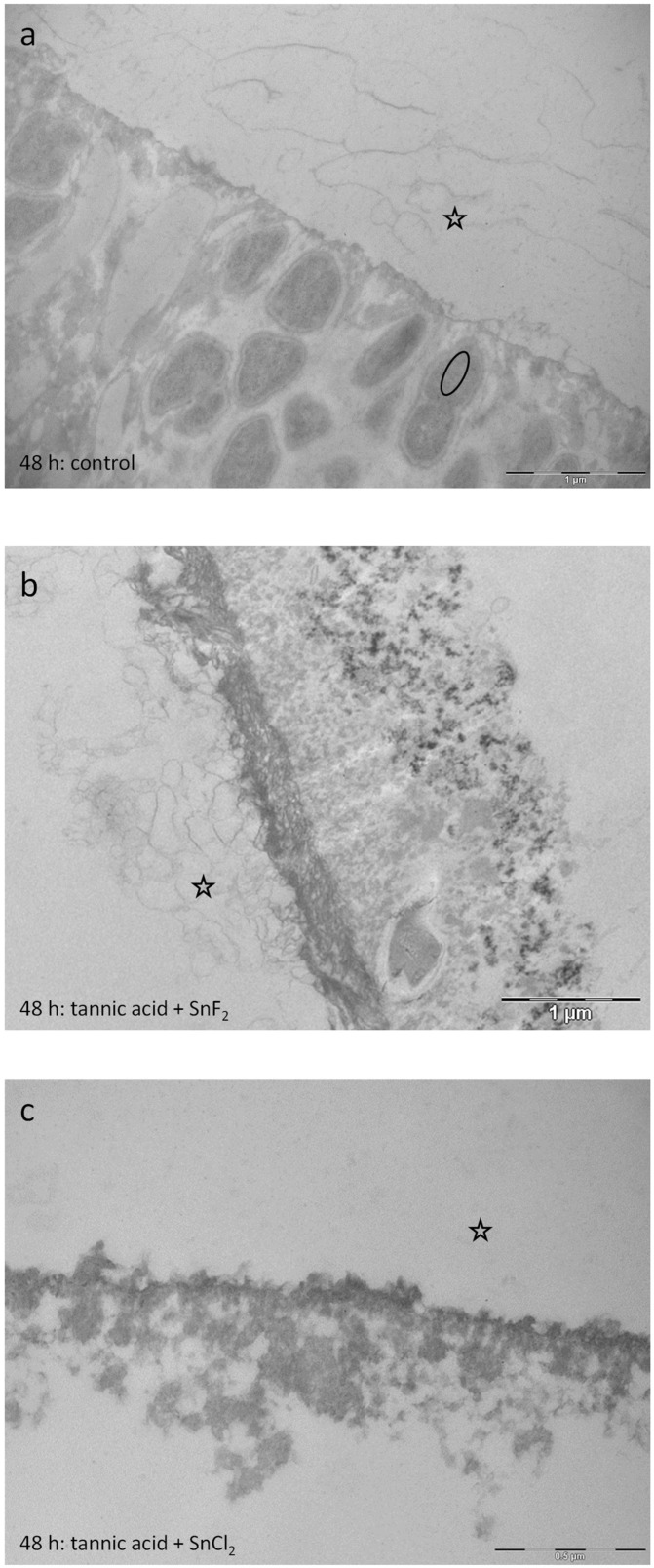
Representative TEM images visualize the pellicle’s ultrastructure. Intraoral exposure time: 48 h. (**a**) Typical sample of a control pellicle layer (no rinsing) consisting of an electron-dense basal layer and less electron-dense globular protein compounds above. Intact bacteria are detectable (oval). (**b**) Rinsing with tannic acid and stannous fluoride for 10 min, pH 3.2. Rinsing with this combination leads to an electron-dense basal pellicle layer with protein infiltrations below. (**c**) Rinsing with tannic acid and stannous chloride for 10 min. pH 2.45. Protein infiltrations are detectable below the basal layer of the pellicle. The former enamel site is marked by a hollow star. Scale bar in the right corner: (**a**,**b**): 1 µm; (**c**): 0.5 µm.

After 48 h intraoral exposure, the control samples showed a typical dense basal layer and less electron-dense globular protein compounds above. Bacteria are partly detectable as ghost cells with a collapsed bacterial cell wall. Rinsing with the combinations leads to pellicle layers with protein infiltrations below (Figure 17b,c). These results are comparable to the 8 h samples (Figure 15a–d). Consequently, the demineralization of the tooth surface due to the low pH seems to be an early effect directly after rinsing with the solution.

EDX-analysis was performed additionally to confirm the observations of the TEM images after rinsing with stannous ions. Figure 14e,f shows dark spots only in samples that were rinsed with stannous ion-containing solutions. Expectorate samples were analyzed, and traces of Sn were detected by EDX analysis in the pellicle samples treated with SnF_2_, the combination of tannic acid and SnCl_2_, and the combination of tannic acid and SnF_2_.

## 4. Discussion

The influence of combined mouth-rinsing solutions composed of tannic acid and stannous ions was investigated on the protective properties of the in situ pellicle for the first time in the present study. The aim of this clinical–experimental study was to examine the effect of these combinations on the bacterial colonization of the pellicle, as well as their influence on enamel erosion protection. The evaluation of the fluorescence microscopic procedures after a single rinse with the tested mouthrinses showed a significant reduction in bacterial adhesion as well as glucan synthesis (Figure 1, Figure 2, Figure 3, Figure 4 and Figure 5). Several previous studies have already shown the inhibitory effect of both stannous ions and polyphenol extracts on bacterial adherence [11,29]. The exact biochemical processes that contribute to the positive effects of these substances are not yet well-understood. There are various hypotheses about the underlying mechanisms of action.

### 4.1. Antibacterial Mechanisms of Tannin

Tannic acid can inhibit the peroxidation of membrane lipids. This leads to an antiviral and anti-mutagen effect of this phenolic substance [39]. Furthermore, tannic acid interacts with molecules and metal ions within bacteria. This reaction leads to an increase in the permeability of the cell membrane, a destruction of the stability of the membrane, and a change in the protein–lipid ratio [3,14,39]. In the present study, damaged bacterial cells were detected after rinsing with tannic acid and stannous fluoride in the expectorate samples via TEM. Moreover, denaturation of pellicle proteins leads to impaired interaction between microorganisms and bacterial receptors. In addition, microbial enzymes can be inhibited. This results in antibacterial activity and impaired microbial adhesion [3,14]. In the run-up to the present study, it was not clear if tannic acid binds to the stannous ions within the rinsing solutions and thereby acts as an inhibiting chelating agent. Consequently, it could not be ruled out whether polyphenol–stannous ion complexes are formed, which either bind to the pellicle surface, remain in the oral fluids, or bind to the mucosal pellicle and finally get swallowed, lowering the effect of the combined rinsing solutions. It is known that saliva and the oral cavity have substantial effects that modulate interactions significantly. Therefore, the study has been designed to examine possible reactions between tannic acid and stannous ions in the oral environment, with potential effects of salivary proteins and peptides [28]. Consequently, rinsing with tannic acid first and stannous fluoride or chloride second was performed, as well as the opposite sequence with stannous fluoride or chloride first and tannic acid second. Our aim was to examine if one or the other component was acting stronger in reducing adherent bacteria and erosive attacks on the tooth surface in situ, since they have different binding mechanisms to the pellicle layer. The results of fluorescence microscopy showed no statistical difference between the groups with different sequence procedures regarding a reduction in bacterial adherence and glucan formation. Interestingly, the sequence tannic acid first and stannous chloride second showed a significantly lower reduction in bacterial adherence to the tooth surface than the other sequences and the combinations. Since there was no significant difference between the other sequences and combinations, the experiments regarding the erosion-protective effect were continued with the combinations for practical reasons. It was shown that the combinations demonstrated significantly better results in reducing bacterial adherence to the tooth surface and showed significantly higher erosion-protection properties than rinsing with tannic acid only. It can therefore be assumed that tannic acid does not inhibit the effect of stannous ions.

### 4.2. Protective Mechanisms of Stannous Ions

Stannous ions are also capable of undergoing various reactions within the oral cavity. Stannous fluoride is known as a caries-preventive agent as well as an effective plaque inhibitor [29,40]. Stannous ions adsorb to the surface of microorganisms and thereby influence the metabolism within the bacterial cell. In addition, thiol groups of bacterial enzymes can be oxidized, leading to changes in microbial adhesion [41,42]. Another explanation for the plaque-inhibiting effects is the inhibition of extracellular bacterial enzymes. As a result, bacteria can no longer form an extracellular polysaccharide matrix, which makes adherence more difficult [43,44]. The positive effect of stannous ions on erosion-protection can be attributed to incorporation into the enamel and pellicle layer resulting in a metallic layer on the tooth surface that protects against acids [26,27]. It was assumed that this effect seems to be enhanced when compared with fluoride ions [27,31]. However, our results could not confirm this statement. The use of stannous fluoride and chloride as monosubstances showed no significant differences regarding the effect of reducing bacterial adherence or improving erosion protection. This is in line with previous in situ results [26,29]. Nevertheless, the fluoride ion seems to play a crucial role in the effect when combined with polyphenols like tannic acid.

### 4.3. Synergistic Effects

The results of the present study have shown that the combination of stannous fluoride and tannic acid yielded significant effects regarding the reduction in bacterial adherence and erosion protection. These findings could have a strong clinical impact in formulating new preventive agents and dentifrices. In comparison to combinations with stannous chloride, the fluoride ion seems to have a synergistic effect within these combinations. The visual examination via TEM certainly gave more insights into the mechanisms of tannic acid and stannous ions. Sequential rinsing with stannous fluoride first and tannic acid second leads to a pronounced electron-dense pellicle layer and resulted in the most intense density in the present experiments (Figure 16e,f). In addition, rinsing with tannic acid second had intense tanning effects on the pellicle layer, forming a protein layer that showed increased density and thickness. The opposite rinsing order—tannic acid (10 min) followed by rinsing with stannous fluoride (1 min)—also led to significant effects on the modification of the pellicle layer’s ultrastructure (Figure 16a,b). The pellicle appeared electron-dense and thicker compared to the control samples. Nevertheless, the effect with stannous fluoride first and tannic acid second was significantly stronger (Figure 16e,f). The combination of tannic acid and stannous fluoride showed less pronounced effects compared to single rinsing with tannic acid or stannous fluoride (less pellicle density and thickness). Conclusively, we can assume that stannous ions can be incorporated to a higher amount when hitting the enamel surface first. Otherwise, protein–polyphenol complexes on the surface might hamper the interaction. The expectorate samples further suggest that tannic acid chelates stannous ions. These complexes adhere further to the pellicle layer, leading to incorporation into the pellicle ultrastructure. The deposition of stannous ions seems to take place in the first hours after rinsing, since stannous ions were incorporated into the lower layers of the pellicle (Figure 14e–h). Many external agents like HAP particles have shown initially good results, adhering to the tooth surface, but showed no substantivity. In contrast, the present study demonstrated that combinations of stannous ions and polyphenolic solutions such as tannic acid show a high substantivity on the tooth surface, with sustainable effects after 48 h. Tannic acid can complex metal ions and additionally shows a high affinity to protein structures. We assume that tannic acid chelates stannous ions and adheres further to the pellicle layer, leading to an incorporation into the pellicle ultrastructure. TEM images of single rinsing with stannous chloride showed the so-called subsurface pellicle—protein infiltrations that cover small, eroded areas—due to the low pH of stannous chloride [29]. In contrast to these findings, subsequent rinsing with stannous chloride first and tannic acid second lead to an offsetting of the negative effects from stannous chloride rinsing (Figure 16g,h). No protein infiltrations were observed below the pellicle layer. Since the stannous chloride solution still had a pH 2.5, rinsing with tannic acid seems to nullify the demineralizing effect of the stannous chloride solution. Green tea is a natural example for a food product that contains plant-derived polyphenols and fluorides. The measured fluoride amount in green tea infusions is approximately 0.26–4.09 ppm of fluoride [45]. Nevertheless, several studies assume that catechins, rather than fluoride, are the most important factor in the caries-preventive effects of green tea [46,47,48]. Han et al. showed that the combination of fluoride and catechins had synergistic antimicrobial effects in vitro [32]. In the presence of fluoride, both non-galloylated catechins, which showed no ability to inhibit acid production individually, and galloylated catechins showed significantly enhanced inhibitory effects on *S. mutans* acid production under acidic conditions in vitro (pH 5.5) [32]. In the present study, the measured fluoride amount of the rinsing solutions and combinations with stannous fluoride was much higher (500 ppm). Since the combination of tannic acid and stannous fluoride showed better results than rinsing with tannic acid and stannous chloride, we conclude that the applied concentration of fluoride ions provides additional synergistic effects. So far, there are a few in vitro studies evaluating the effect of combinations of polyphenols and fluorides [5,49], but there are some limitations. Baumann et al. used commercially available rinsing solutions with extracts of grape seeds and cranberries, sodium fluoride, peppermint oil, and xylitol (2 g/L polyphenol content), as well as a commercially available stannous fluoride solution with anethole, anise oil, mint extract, saccharine, and menthol in vitro [5]. The stannous fluoride solution served as comparison, since it represents the current gold standard of erosion-protective rinsing solutions [23]. The erosive challenge was performed at a rather mild pH of 3.6 [5]. It was shown that the grape seed extract with sodium fluoride and additives, as well as the cranberry extract with sodium fluoride and additives, performed comparably to the commercially available stannous fluoride-solution. The same study group investigated synergistic effects between plant extracts and fluoride to protect against enamel erosion in vitro [49]. Carvallho et al. examined the polyphenol-rich plant extracts of grape seed, grapefruit seed, and blueberry and combined them with 500 ppm fluoride, deriving either from sodium fluoride or from a mixture of sodium fluoride and amine fluoride. In addition, they also used a commercially available stannous fluoride solution to compare with [49]. Carvallho et al. showed that the combination of fluoride and polyphenols led to a higher erosion-protective effect than the solution containing only fluoride or only polyphenols [49]. The present study also investigated possible synergistic effects. Though, there are differences. It was previously shown that rinsing solutions with fluorides provided greater protection against erosive tissue loss when used in situ than used in vitro [33]. Reasons are the complex maturation processes of the pellicle, since there is continuous remodeling. Additionally, the physiological pellicle contains not only salivary proteins but also proteins from the sulcus fluids and components of epithelial cells or from bacteria. Therefore, in situ studies allow the investigation of modulating effects from experimental solutions to the pellicle under physiological conditions. Another crucial factor is the availability of proteins, when testing polyphenolic substances, since their protective effect is based on protein-crosslinking. It was demonstrated that polyphenols show substantivity in the oral cavity by these protein-binding-mechanisms [13,50]. These effects cannot be covered under in vitro conditions. The present results showed that the reducing effect on bacterial adherence was still significant after rinsing with the combination of tannic acid and stannous fluoride and performing the experiments after 48 h of oral exposition. This could not be confirmed for the combination of tannic acid and stannous chloride. Another difference in the present study is the use of monosubstances instead of commercially available rinsing solutions. This ensures that possible effects can be attributed to the single components. Additionally, we decided to perform the experiments with stannous fluoride in combination with the tannic acid, since it is the gold standard and showed significantly better results regarding erosion protection and caries prevention than other fluoride-bound cations [26,29]. Nevertheless, there are some limitations of the present study: the experiments simulating clinical erosion were performed ex vivo, since more participants were willing to take part in the study. However, as a result, salivary buffer capacity, clearance rate, salivary dilution, and provision of further salivary minerals could not be considered [51]. Additionally, bovine enamel samples (*n* = 796) were chosen to generate enough samples with high quality and homogeneity for all the experiments [52].

## 5. Conclusions

The present study shows that stannous fluoride and stannous chloride significantly improve the pellicle’s caries- and erosion-protective properties when compared to rinsing with tannic acid alone. Mouthrinses containing stannous fluoride, or the combination of tannic acid and stannous fluoride, were superior to the combination of tannic acid and stannous chloride. The combinations showed a high substantivity on the tooth surface, with sustainable effects after 48 h. Thereby, the deposition of stannous ions seems to take place in the first hours after rinsing, since stannous ions were incorporated into the lower layers of the pellicle. Consequently, bacterial adherence was affected. Therefore, we assume a synergistic effect of tannic acid and fluoride ions that can modify the pellicle-protective properties against erosion and caries after 8 h and 48 h in situ. Stannous fluoride, or the combination of tannic acid and stannous fluoride, should be strongly considered for the development of new preventive agents.

## Figures and Tables

**Table 1:** Solution volume, weighed quantity, and measured pH value of the solutions used in the present study.

Pure Substance	Solution Volume	Weighed Quantity	Measured pH Value
Stannous fluoride (Honeywell Speciality Chemicals GmbH, Seelze, Germany)	8 mL	16.5 mg dissolved in double-distilled water	4.5
Stannous chloride (Honeywell Speciality Chemicals GmbH, Seelze, Germany)		23.768 mg dissolved in double-distilled water	2.5
Tannic acid (pharmaceutically pure, Caelo, Caesar & Lorentz GmbH, Hilden, Germany)		13.6 mg dissolved in double-distilled water	3.9
Combination of tannic acid and stannous fluoride Relation 1:1		6.8 mg TA and 8.25 mg SnF_2_ dissolved in double-distilled water	3.2
Combination of tannic acid and stannous chloride Relation 1:1		6.8 mg TA and 11.884 mg SnCl_2_ dissolved in double-distilled water	2.45

## Data Availability

Data is contained within the article.

## Abbreviations

The following abbreviations are used in this manuscript:

SnF_2_	Stannous Fluoride
SnCl_2_	Stannous chloride
TEM	Transmission electronic microscopy
EDX	Electron dispersive x-ray
DAPI	4′,6-Diamidin-2-phenylindol
Con A	Concanavalin A
